# Effects of NSAIDs on the Inner Ear: Possible Involvement in Cochlear Protection

**DOI:** 10.3390/ph3051286

**Published:** 2010-04-27

**Authors:** Tomofumi Hoshino, Keiji Tabuchi, Akira Hara

**Affiliations:** Department of Otolaryngology, Graduate School of Comprehensive Human Sciences, University of Tsukuba, Tsukuba, 1-1-1 Tennodai, Tsukuba 305-8575, Japan; E-Mails: tomofumi@tara.tsukuba.ac.jp (T.H.); haraakir@md.tsukuba.ac.jp (A.H.)

**Keywords:** NSAIDs, cochlea, cyclooxygenase, lipoxygenase

## Abstract

Cyclooxygenase and lipoxygenase, two important enzymes involved in arachidonic acid metabolism, are major targets of non-steroidal anti-inflammatory drugs (NSAIDs). Recent investigations suggest that arachidonic cascades and their metabolites may be involved in maintaining inner ear functions. The excessive use of aspirin may cause tinnitus in humans and impairment of the outer hair cell functions in experimental animals. On the other hand, NSAIDs reportedly exhibit protective effects against various kinds of inner ear disorder. The present review summarizes the effects of NSAIDs on cochlear pathophysiology. NSAIDs are a useful ameliorative adjunct in the management of inner ear disorders.

## 1. Introduction

Non-steroidal anti-inflammatory drugs (NSAIDs) are some of the most commonly used drugs in daily clinical practice. They are usually used as painkillers, antipyretics, *etc.*. In the arachidonic cascade, membrane phospholipids are metabolized into various mediators. Classic NSAIDs inhibit activity of cyclooxygenase (COX) that is responsible for the cyclic endoperoxide of prostaglandin (PG) G2 and PGH2. COX is divided into three subgroups: COX-1, COX-2, and COX-3 [1,2]. COX-1 is constitutively expressed in many mammalian tissues. On the other hand, COX-2 is induced in response to various stimuli, such as cytokines, bacterial lipopolysaccharide, and growth factors [3,4]. Furthermore, high expression of COX-2 is typical for prenatal period [5,6]. COX-3 is a splicing variant derived from the Cox1 gene. This protein is expressed in the central nervous system and involved in sensitive neuronal pathway [2]. Use of COX-2 selective NSAISDs reduces incidence of gastrointestinal side effects. However, typical complications induced by COX-2 inhibitors were also found, such as cardio-vascular complications. Therefore, it is important to clarify the characteristics of the subtypes of NSAIDs and their usage.

The arachidonic cascade is made up of several enzymes, such as a phospholipase A2 (PLA2), a lipoxygenase (LOX), and a monooxygenase pathway, as well as COX. In the first step of the arachidonic cascade, arachidonic acid (AA) is generated from membrane phospholipid precursors by PLA2. The next stage of this cascade involves two main pathways: the COX pathway that produces prostaglandins (PGs) and thromboxanes (TXs), and the LOX pathway that produces leukotrienes (LTs). In the monooxygenase pathway, AA is primarily metabolized by cytochrome P450 (CYP) enzymes to hydroxyeicosatetraenoic acids (HETEs) and epoxyeicosatrienoic acids (EETs) [7]. In this review, we summarized the involvement of COX and COX inhibitors in cochlear pathophysiology. The effects of inhibitors of other arachidonic cascade enzymes on the cochlea are also reviewed.

## 2. Ototoxicity Induced by NSAIDs

NSAIDs are used in the clinical practice of various departments; e.g., in the otolaryngological department, they are used as analgesic agents for otitis media, sinusitis, tonsillitis, and other diseases. Although no problem occurs due to their usage in most cases, side effects of NSAIDs do occur in some cases. Common side effects are gastric mucosal injury, renal function impairment, allergic reactions, and cardio-vascular complications. In addition to these side effects, high-dose treatments of NSAIDs, especially aspirin and its active metabolite salicylate, occasionally induce ototoxicity, including tinnitus, and hearing loss [8,9,10]. In such cases, tinnitus is often the first subjective symptom. Subsequently, mild to moderate hearing loss, usually reversible tends to occur. The severity of hearing loss is reportedly correlated with the plasma salicylate level [10].

Many animal studies have been conducted to clarify the mechanisms of the otological side effects of NSAIDs. Otoacoustic emission (OAE) is a useful test to monitor outer hair cell function. In animal examinations, a reduction of the OAE level was observed after the administration of high-dose salicylate [11,12]. Additionally, thresholds of both an eighth nerve compound action potential (CAP) and an auditory brainstem response (ABR), indicators of the hearing level, decreased transiently after high-dose sodium salicylate medication [13,14]. The perilymphatic perfusion of a high concentration of salicylate decreased the CAP threshold in guinea pigs, inducing mild to moderate hearing loss [15].

Although the systemic administration of NSAIDs does not affect cochlear movement because the inhibitory concentration is considered to be much higher than the physiological level achieved by systemic administration, high-dose NSAID medication inhibits cochlear movement, which can be measured by laser interferometry [16,17]. On the other hand, the endocochlear potential (EP), an indicator of the function of the stria vascularis, did not change after NSAID treatment [18,19,20]. All these data obtained from animal studies seem to reflect the impairment of the active process of the cochlea, the mechano-sensory function of the outer hair cells, caused by high-dose NSAIDs. Regarding the morphological aspect, electron microscopic examinations have demonstrated that minor changes in the stereocilia of hair cells occur after high-dose NSAID treatment, although light microscopic examinations did not show any abnormality of stereocilia [21]. This phenomenon is also observed in humans. Namely, NSAID ototoxicity also reportedly leads to a reduction of the OAE level [22,23].

Although mild to moderate sensorineural hearing loss induced by salicylate has been attributed to impaired sound amplification by outer hair cells through its direct action on their motility, there is a disparity in salicylate concentrations between clinical and animal studies, *i.e.*, extremely high extracellular concentrations of salicylate are required to induce a significant reduction of electromotility in animal studies. Wu *et al.* [24] recently reported that the clinical concentration range of salicylate caused concentration-dependent and reversible reductions in IK,n (KCNQ4) and the subsequent depolarization of outer hair cells. They suggested that this reversible IK,n reduction might cause the otologic side effects of salicylate.

In addition to the reports that an excessive amount of salicylate induces the dysfunction of hair cells, as described above, recent studies have demonstrated that high doses of salicylate also induce the degeneration of cochlear spiral ganglion neurons [25], and impair auditory neural activity of the cochlea [26]. It has been demonstrated that arachidonic acid potentiates NMDA receptor currents [27]. The spiral ganglion neurons express NMDA receptors [28]. Although fast excitatory synaptic neurotransmission is predominantly mediated by AMPA receptors in the cochlea [29,30], 

Guitton *et al*. [31] suggested that salicylate induced tinnitus through the activation of these cochlear NMDA receptors. Furthermore, salicylate induces the abnormal excitability of neurons in the brainstem, subcortical area, and auditory cortex [32,33,34]. Based on recent evidence from both evoked potentials and neuron-pair synchrony measures, it is unlikely that tinnitus is the expression of a set of independently firing neurons, and more likely that it is the result of a pathologically increased synchrony between sets of neurons [35]. Thus, in addition to the impairment of outer hair cells, changes in the excitability of auditory peripheral or central neurons may be the cause of the otological side effects of salicylate.

Cazals [10] reviewed the existence of numerous types of metabolic interference by salicylate: the inhibition of several enzymes including NADPH oxydase, phospholipase C, cholesterol ester synthase, and ATPase, the inhibition of antigen-antibody interactions, insertion into membranes and interference with ion transport, uncoupling of oxidative phosphorylation, hyperglycemia, activation of heat shock transcription factor, and the inhibition of free radicals in addition to inhibition of prostaglandin synthesis. After Cazals’s review, several studies demonstrated the enhanced expression of TRP1 [36], prestin [37] and brain-derived neurotrophic factor (BDNF) [38,39], and morphological changes [40,41]. Prestin is a motor protein involved in the motility of outer hair cells [42]. Although functional testing of the cochlea showed that the impairment of outer hair cell motility transiently occurred after high-dose salicylate treatment, recent research demonstrated that the long-term administration of salicylate inversely increased the expression of prestin in the cochlea [37,43]. Although some evidence has been accumulated, the precise origin and mechanism(s) of NSAID-induced ototoxicity has not been fully clarified.

## 3. The Protective Effects of NSAIDs against Cochlear Injury

Although we reviewed the ototoxicity of NSAIDs in the previous section, we will describe their protective effects on cochlear injury in this section. Various protective effects of NSAIDs on cochlear injury have been reported in animal studies. Several NSAIDs reportedly exhibit protective effects on the inner ear against acoustic injury in rodents [44,45]. Furthermore, salicylate protects the cochlea against ototoxicity induced by aminoglycoside or cisplatin [46,47,48]. It has been demonstrated in animals subjected to acoustic injury that there is a window of opportunity for rescue from noise-related trauma by delayed pharmacological intervention with salicylate after the onset of this type of injury [49].

Regarding the subtypes of COX, Stjernschantz *et al.* [50] reported that COX-1 is expressed in the cochlea, but COX-2 is not. On the other hand, Ziegler *et al.* [51] demonstrated that both COX-1 and COX-2 are expressed in several types of inner ear cells. Although COX-2 is generally known to be an inducible enzyme responding to various stimuli, Heinrich *et al*. [52] demonstrated that COX-2 constitutively exists in the normal inner ear and that sound exposure down-regulates COX-2 expression in the inner ear. This finding suggests that COX-2 may show a different response pattern on receiving stimuli in the cochlea compared to other organs. On the other hand, little is known about LOX expression. However, there is a report that mRNA of LOX was detected in an organ of Corti-derived immortalized cell line [53]. 

In our previous paper [54], the differences of NSAIDs were focused in terms of inhibitory enzymes regarding the protective effect. Namely, it was examined which subtypes of NSAID protected the cochlea against acoustic injury. For this purpose, the effects of non-selective NSAID (indomethacin), semi-selective COX-2 inhibitor (meloxicam), selective COX-2 inhibitors (SC58125 and CAY10404), and LOX inhibitor (nordihydroguaiaretic acid) were tested in mice subjected to acoustic overexposure of 128 dB SPL (sound pressure level) for 4 hours. All the tested non-selective NSAID and LOX inhibitors protected the cochlear hair cells against acoustic injury, whereas COX-2 semi-selective and selective inhibitors did not exhibit any protective effect [54]. Based on this finding, it is assumed that it is important to consider subtypes of NSAID for cochlear protection [54].

PLA2, the first-step enzyme of the arachidonic cascade, is reportedly involved in the generation of cochlear ischemic [55] and acoustic [56] injury. Although quinacrine, a general PLA2 inhibitor, ameliorated the cochlear ischemia-reperfusion and acoustic injury [55,56], the precise protective mechanisms are largely unknown. As described above, the protective effects of inhibitors of COX-1 and LOX as well as PLA2 have been demonstrated in various kinds of cochlear injury. Further studies on the arachidonic cascade in the cochlea and its inhibitors (or modulators) will shed light on the understanding of the generation mechanisms of various cochlear injuries.

In clinical practice, sensorineural hearing loss often arises from cochlear impairment due to several causes: some kinds of drug including aminoglycoside antibiotics and anti-cancer drugs, loud sounds, ischemia, and aging. Several researchers have demonstrated that glucocorticoids exhibit protective effects against cochlear injuries in animal studies [56,57,58]. Considering that glucocorticoids regulate the arachidonic cascade by inhibiting the PLA2 [59], it can be speculated that one of the protective mechanisms of glucocorticoids against cochlear injury is derived from modulation of the arachidonic cascade, although glucocorticoids are multi-functional agents.

## 4. Protective Mechanisms of NSAIDs in Cochlear Injury

Little is known regarding the protective mechanisms of NSAIDs against inner ear injury. However, given that NSAIDs exhibit both anti-inflammatory and anti-oxidant actions, these actions might also be related to their protective effects in the inner ear.

Regarding the anti-inflammatory actions of NSAIDs, several products of the arachidonic cascade are related to inflammation. For example, PGs are very well-known inflammatory agents, which have potent effects on vasodilatation and vascular permeability [60]. Additionally, it has been demonstrated that the over-production of TXs and LTs induces inner ear injury [61,62]. Based on these findings, there is a possibility that NSAIDs are able to protect against inner ear injury by inhibiting the over-production of these metabolites.

Another possible mechanism of cochlear protection is the anti-oxidant actions of NSAIDs. It has been reported that reactive oxygen species (ROS) are involved in several inner ear injuries including drug-mediated ototoxicity [63,64], loud sound [65,66,67,68], ischemia [69,70,71], and aging [72]. Dinis *et al.* [73] firstly demonstrated that salicylate was a radical scavenger. In regard to ROS production via the arachidonic cascade, ROS are produced during the conversion of PG-G2 to PG-H2 in the COX pathway and hydroperoxy-eicosatetraenoic acid to hydroxy-eicosatetraenoic acid in the LOX pathway [74]. COX and LOX inhibitors, namely NSAIDs, can therefore block ROS production.

Furthermore, in addition to the anti-inflammatory and anti-oxidant actions of NSAIDs, salicylate is known to regulate the transcriptional factor nuclear factor kappa B (NF-κB), thereby intervening in an apoptotic pathway [75,76]. The translocation of NF-κB from the cytosol to nucleus increases in the presence of ototoxic stimuli including exposure to an excessively loud sound [77], cisplatin [78], and aminoglycosides [79]. Salicylate has a capacity to inhibit the translocation of NF-κB to the nucleus based on its action on IκB kinase [76], and may thus intervene in the apoptotic pathway. These mechanisms have also been proposed to explain the protective effect of NSAIDs. 

## 5. Conclusions

The usage of NSAIDs at excessively high doses will induce inner ear disturbances, causing tinnitus and mild to moderate sensorineural hearing loss. These otological side effects are often transient and reversible after the cessation of NSAID consumption. Although these precise mechanisms of these side effects have not been fully clarified, impairment of the outer hair cell function seems to be one of the main causes of side effects. Another possible mechanism of these otological side effects of NSAIDs is their excitation of the central auditory nervous system. 

On the other hand, recent studies have demonstrated that NSAIDs exhibit protective effects on cochlear injuries in animal studies. Although glucocorticoids are widely used for inner ear disorders in humans today, the treatment results are not fully satisfactory, and, thus, there is presently no effective therapy for inner ear hearing loss. Basic experimental findings suggest that NSAIDs are potential agents for inner ear disturbances in humans. Further investigations regarding NSAIDs are necessary to clarify the mechanisms of their side effects and their potential protective actions. 
